# Methylglyoxal improves zirconium stress tolerance in *Raphanus sativus* seedling shoots by restricting zirconium uptake, reducing oxidative damage, and upregulating glyoxalase I

**DOI:** 10.1038/s41598-023-40788-0

**Published:** 2023-08-21

**Authors:** Yoneal Bless, Linda Ndlovu, Esihle Gcanga, Lee-Ann Niekerk, Mbukeni Nkomo, Olalekan Bakare, Takalani Mulaudzi, Ashwil Klein, Arun Gokul, Marshall Keyster

**Affiliations:** 1 Environmental Biotechnology Laboratory, Department of Biotechnology, University of the Western Cape, Bellville, 7535 South Africa; 2 Department of Biotechnology, Life Science Building, University of the Western Cape, Bellville, 7535 South Africa; 3 Plant Omics Laboratory, Department of Biotechnology, University of the Western Cape, Bellville, 7535 South Africa; 4 Department of Plant Sciences, Qwaqwa Campus, University of the Free State, Phuthadithjaba, 9866 South Africa; 5 DST-NRF Centre of Excellence in Food Security, University of the Western Cape, Bellville, 7530 South Africa

**Keywords:** Plant sciences, Plant signalling, Plant stress responses

## Abstract

*Raphanus sativus* also known as radish is a member of the Brassicaceae family which is mainly cultivated for human and animal consumption. *R. sativus* growth and development is negatively affected by heavy metal stress. The metal zirconium (Zr) have toxic effects on plants and tolerance to the metal could be regulated by known signaling molecules such as methylglyoxal (MG). Therefore, in this study we investigated whether the application of the signaling molecule MG could improve the Zr tolerance of *R. sativus* at the seedling stage. We measured the following: seed germination, dry weight, cotyledon abscission (%), cell viability, chlorophyll content, malondialdehyde (MDA) content, conjugated diene (CD) content, hydrogen peroxide (H_2_O_2_) content, superoxide (O_2_^*•−*^) content, MG content, hydroxyl radical (**·**OH) concentration, ascorbate peroxidase (APX) activity, superoxide dismutase (SOD) activity, glyoxalase I (Gly I) activity, Zr content and translocation factor. Under Zr stress, exogenous MG increased the seed germination percentage, shoot dry weight, cotyledon abscission, cell viability and chlorophyll content. Exogenous MG also led to a decrease in MDA, CD, H_2_O_2_, O_2_^*•−*^, MG and **·**OH, under Zr stress in the shoots. Furthermore, MG application led to an increase in the enzymatic activities of APX, SOD and Gly I as well as in the complete blocking of cotyledon abscission under Zr stress. MG treatment decreased the uptake of Zr in the roots and shoots. Zr treatment decreased the translocation factor of the Zr from roots to shoots and MG treatment decreased the translocation factor of Zr even more significantly compared to the Zr only treatment. Our results indicate that MG treatment can improve *R. sativus* seedling growth under Zr stress through the activation of antioxidant enzymes and Gly I through reactive oxygen species and MG signaling, inhibiting cotyledon abscission through H_2_O_2_ signaling and immobilizing Zr translocation.

## Introduction

*Raphanus sativus* seeds are used to produce biofuels^1^ and the seedlings can be eaten raw as microgreens or added to cooked dishes^2^. Roots are consumed as salad, cooked, pickled, canned and dried. The leaves can be cooked as a vegetable for human consumption or used as highly digestible animal feed^2^. The demand for *R. sativus* as a human food source remains moderate but there is a growing need for production as animal feed during the winter season^3^. However, *R. sativus* production is restricted by heavy metal stress such as cadmium (Cd)^4^, lead (Pb)^5^, arsenic (As)^6^, chromium (Cr)^7^, mercury (Hg)^8^, antimony (Sb)^9^, and nickel (Ni)^10^. But, no data exist in literature about the effect of zirconium (Zr) on *R. sativus*.

Zr is ranked the 20th most common element in the Earth’s crust and is generally immobile in soils due to low water (H_2_O) solubility^11^. Australia and South Africa are the top two mining producers of Zr worldwide but Zr mining also occur in Kenya, Senegal, Mozambique, Ukraine, India and Indonesia^12^. Due to excessive mining, Zr can seep from soils into rivers and the continuous build-up can lead to pollution and contamination of the environment^13^. Therefore, Zr is characterized as a potentially hazardous chemical in a similar class as Cr, As, thallium, and Hg^14^. Zr levels in the Earth’s crust fluctuates between 32 and 850 μg g^−1^ and Zr mostly exist as zircon (ZrSiO_4_) and baddeleyite (ZrO_2_)^15^. Elevated Zr content taken up by plants from the soil reduced the root and shoot growth of *Triticum aestivum*^16^ and *Brassica rapa*^17^ even though the transport mechanism is still unknown. Plants treated with high levels of Zr display growth stunting and chlorosis due to the degradation of chlorophyll^16^. The exact mode of Zr induced reduction in plant growth or modification of enzymes is not well understood but Shahid et al.^15^ speculates that Zr toxicity leads to overproduction of ROS (H_2_O_2_, O_2_^•−^ and ·OH), which subsequently incur lipid damage and cell death. CD and MDA are constituents of membrane lipids and serve as important biomarkers of lipid peroxidation in plants under stress^18^. In addition, heavy metal stress can induce the continuous production of a reactive α, β-dicarbonyl ketoaldehyde called MG^19^ which can inhibit cell proliferation and cause protein degradation in plants^20^. The reduction of endogenous MG in plants is an attractive approach to improve plant tolerance to heavy metal stress. MG production under Zr stress has not been investigated to date. Nonetheless, plants use enzymatic defense systems SOD and APX to combat downstream heavy metal toxicity from ROS buildup (H_2_O_2_, O_2_^•−^, and ·OH) and Gly I to reduce toxic MG buildup in affected cells^18^.

Exogenously applying biomolecules which stimulates the plant defense systems is a very good approach for limiting heavy metal-induced damage in plants^21^. MG has emerged as a novel signaling molecule and can be exogenously applied to prime the plant defense system at low concentrations^17^. For example, Li et al.^22^ exogenously applied MG to decrease Zr toxicity in *T. aestivum* seedlings and Li et al.^23^ used exogenous MG to reduced Pb stress toxicity in *T. aestivum* seedlings. However, there are no studies that elucidate the role of MG in *R. sativus* especially under Zr stress. Therefore, the objective of this study is to pretreat *R. sativus* seeds with MG under Zr stress to study possible Zr alleviation mechanisms. Specific objectives include: (1) we measure the effect of exogenous MG on seed germination under Zr stress; (2) we analyse the physiological responses (biomass, chlorophyll content, and cotyledon abscission) of seedling shoots under Zr stress under MG treatment; (3) we measure the biochemical responses (ROS content, lipid peroxidation, antioxidant enzymes and cell death) of R. sativus seedling shoots under Zr stress and MG; (4) we determine the effect of MG on Zr content and translocation from R. sativus seedling roots to shoots. We show that MG protects *R. sativus* against Zr stress through priming of the ROS-antioxidant system and possible interaction with an unknown plant Zr transport system.

## Results

### Exogenous MG improves seedling shoot growth and biomass under Zr stress

**Figure 1:**
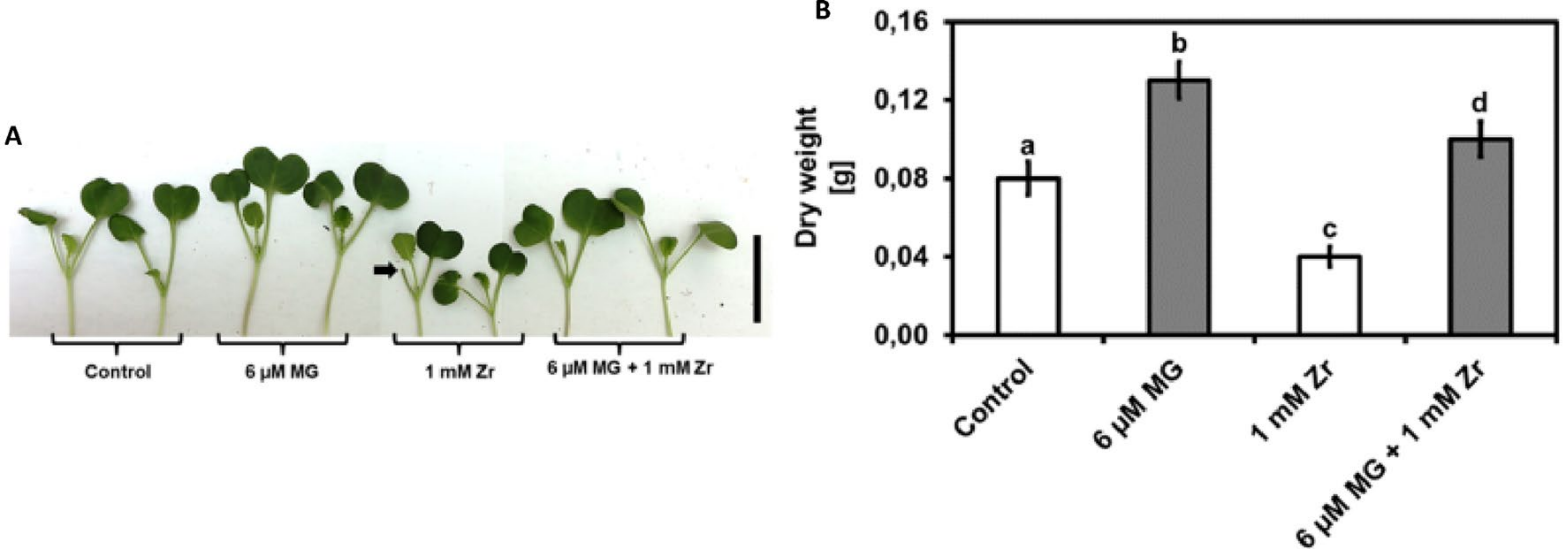
Representative visual image of *R. sativus* seedling growth (**A**) and shoot dry weight (**B**) under control, MG, Zr, MG + Zr treatments. The black arrow shows cotyledon abscission and the black scale bar = 5 cm. Different letters (a, b, c and d) represent the means (± SE) with statistical significance at *p* < 0.05 (Tukey–Kramer test).

We conducted visual inspection on the seedling shoots after the 14-day treatment period and observed an increase in growth and dry weight (65%) when comparing the MG treatment to the control (Fig. 1A,B). Furthermore, we noticed a decrease in seedling growth (Fig. 1A) and dry weight (Fig. 1B) of 50% when comparing the Zr treatment to the control. We also observed widespread cotyledon abscission in the Zr treatment where the seedlings often lost one (Fig. 1) or two cotyledons after the treatment period. We observed similar visual growth for the control and MG + Zr treatment (Fig. 1A) which led to an increase in dry weight of 25% when comparing the two treatments (Fig. 1B). An overall increase in growth and dry weight (150%) as well as no cotyledon abscission was observed in the MG combination treatment with Zr, when compared to the Zr-only-treated seedlings (Fig. 1A,B). This result suggests that MG improves *R. sativus* seedling shoot growth (visually) and biomass with or without Zr stress. Furthermore, MG blocks Zr induced cotyledon abscission under Zr stress.

### Exogenous MG limits Zr stress induced cotyledon abscission in R. sativus

Because we noticed seedling cotyledon abscission on the day of visual inspection and harvesting, we measured the cotyledon abscission percentage across the treatments. We observed no cotyledon abscission in the control, MG, and the MG + Zr combination treatments (Table 1). We only observed cotyledon abscission in the Zr-only data set (Table 1). More than ~ 60% of the seedling shoots lost one or two cotyledons and were scored as cotyledon abscised (Table 1). The data analysis corroborated the visual abscission observation and suggested that Zr causes cotyledon abscission in *R. sativus* and that MG blocks cotyledon abscission under Zr stress.

**Table 1:** Cotyledon abscission (%) in *R. sativus* seedling shoots in response to control, MG, Zr, MG + Zr treatments.

Control	6 µM MG	1 mM Zr	6 µM MG + 1 mM Zr
0.00 ± 0.00^a^	0.00 ± 0.00^a^	63.00 ± 6.00^b^	0.00 ± 0.00^a^

Different letters (a and b) per row indicate the means (± SE) that are significantly different at *p* < 0.05 (Tukey–Kramer test).

### MG improves seedling shoot chlorophyll content under Zr stress

We measured the chlorophyll content in the intact *R. sativus* seedling shoots after the treatment period. Compared to the control, MG treatment led to a 72% increase in chlorophyll *a* content, Zr treatment led to a 37% reduction in chlorophyll *a* content and the MG + Zr combination treatment led to a 59% increase in chlorophyll *a* content (Table 2). We observed an increase in chlorophyll *a* content of 153% in the MG + Zr combination-treated seedlings when compared to the Zr treatment (Table 2). Furthermore, compared to the control, MG treatment led to no significant difference in chlorophyll *b* content, Zr treatment led to a decrease in chlorophyll *b* content of 41% and the MG + Zr treatment led to no change in chlorophyll *b* content. When we compared the chlorophyll *b* content in the MG + Zr combination-treated seedlings to the Zr treatment, we observed an increase of 87% (Table 2). MG treatment increased the total chlorophyll (*a* + *b*) content by 56% and Zr treatment reduced the total chlorophyll content by 38% when compared to the control, respectively. Furthermore, the MG + Zr treatment increased the total chlorophyll content by 46% when compared to the control and by 136% when compared to the Zr-treated seedlings (Table 2).

**Table 2:** Chlorophyll concentration in *R. sativus* seedling shoots in response to control, MG, Zr and MG + Zr treatments.

Chlorophyll (µg g^−1^)	Control	6 µM MG	1 mM Zr	6 µM MG + 1 mM Zr
*a*	124.53 ± 10.11^a^	214.52 ± 14.32^b^	78.33 ± 5.44^c^	198.56 ± 12.59^b^
*b*	48.51 ± 3.91^a^	55.22 ± 4.22^a^	28.73 ± 5.94^b^	53.86 ± 4.56^a^
*a* + *b*	173.04 ± 14.02^a^	269.74 ± 18.54^b^	107.06 ± 11.38^c^	252.42 ± 17.15^b^

Different letters (a, b and c) per row indicate the means (± SE) that are significantly different at *p* < 0.05 (Tukey–Kramer test).

### MG application reduces ROS content (O_2_^•−^, H_2_O_2_ and ·OH ) and endogenous MG content in seedlings under Zr stress

**Figure 2:**
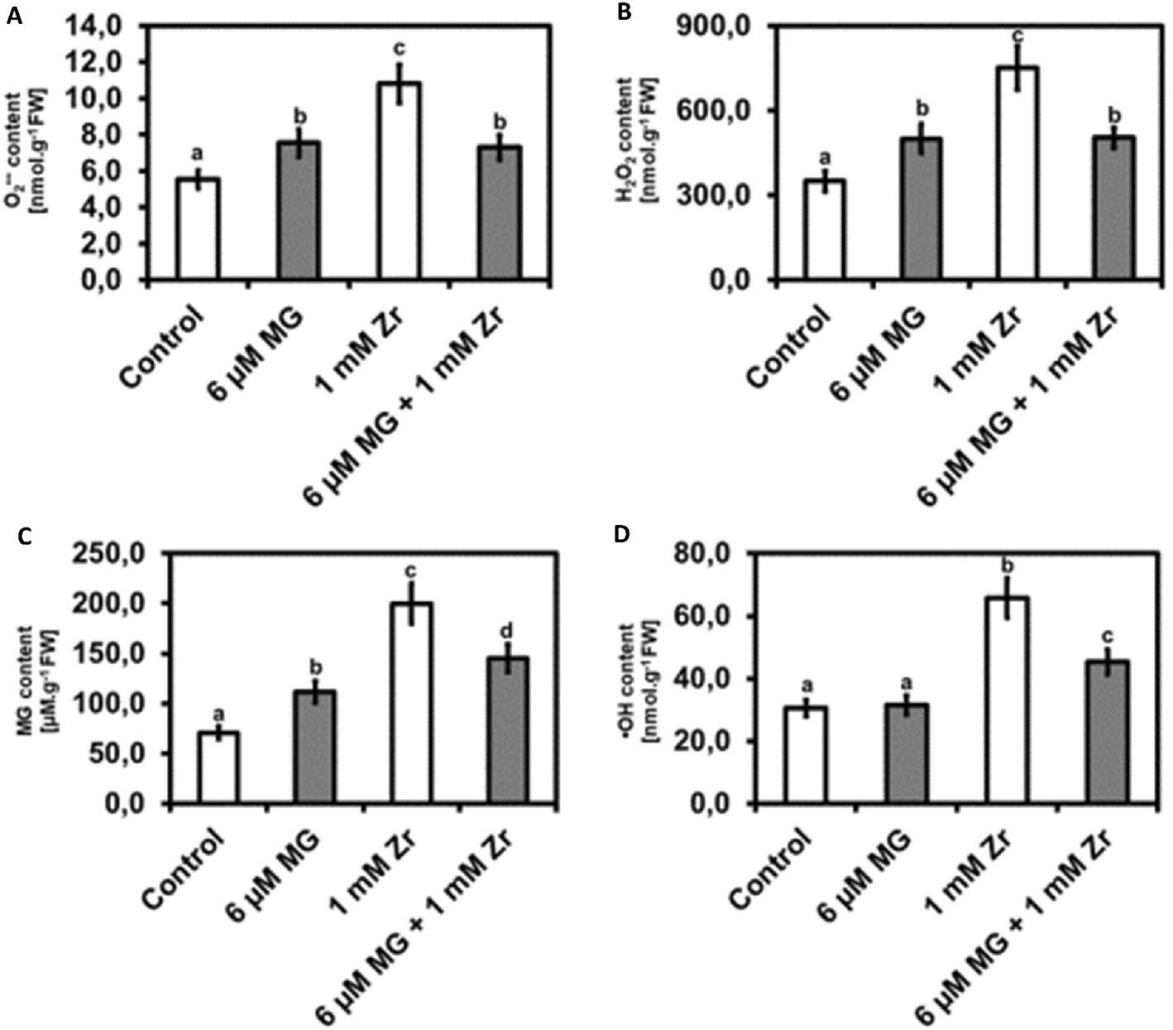
Superoxide content (**A**), hydrogen peroxide content (**B**), MG content (**C**) and hydroxyl radical content (**D**) in *R. sativus* seedling shoots under control, MG, Zr and MG + Zr treatments. Different letters (a, b, c and d) represent the means (± SE) with statistical significance at *p* < 0.05 (Tukey–Kramer test).

To investigate the effect of MG and Zr on *R. sativus* seedling O_2_^•−^ and H_2_O_2_ production, we performed biochemical assays. We observed increases in O_2_^•−^ content in MG-treated seedlings (35%), Zr treatment (94%), and MG + Zr combination treatment (32%) when compared to the control, respectively (Fig. 2A). However, a decrease of 32% was observed in O_2_^•−^ content when comparing the MG + Zr treatment to the Zr-only treatment (Fig. 2A). In the H_2_O_2_ assay, compared to the control, we observed that MG treatment increased the H_2_O_2_ content by 43% and the Zr-treatment increased the H_2_O_2_ content by114%. The MG + Zr combination-treatment increased the H_2_O_2_ content by 44% compared to the control (Fig. 2B). Furthermore, a decrease in H_2_O_2_ content of 33% was observed in MG + Zr combination treatment when compared to the Zr treatment (Fig. 2B). This result indicates that MG application increases both O_2_^•−^ and H_2_O_2_ content in *R. sativus* seedlings and this could be for signaling purposes. Furthermore, Zr application increases both O_2_^•−^ and H_2_O_2_ content in the seedlings and this could be in toxic range which causes negative growth effects.

We also measured the concentrations of MG and ·OH under MG and Zr stress. Compared to the control, MG treatment led to an increase in MG content of 58%, Zr treatment led to an increase in MG content of 183% and the MG + Zr treatment led to an increase in MG content of 106% (Fig. 2C). However, we observed a decrease in MG content of 27% in the MG + Zr combination-treated seedlings when compared to the Zr treatment (Fig. 2C). In the ·OH assay, compared to the control, MG treatment led to no change in the ·OH content, Zr treatment led to an increase in ·OH content of 115% and the MG + Zr com-bination treatment led to an increase in ·OH content of 48% (Fig. 2D). But we observed a decrease in ·OH content of 31% in the MG + Zr combination-treated seedlings when compared to the Zr treatment (Fig. 2D). This result shows that exogenous MG application increased the endogenous MG content but does not change ·OH content in *R. sativus* seedlings.

### Hydrogen peroxide scavenging blocks cotyledon abscission under Zr stress

To test our hypothesis that the substantial increase in H_2_O_2_ could be responsible for the observed induced cotyledon abscission in the Zr-only treatment we treated plants with 1 mM Zr, 5 mM AsA + 1 mM Zr and 5 mM DMTU + 1 mM Zr. AsA and DMTU are two H_2_O_2_ scavengers often used to answer questions about H_2_O_2_ signaling events in plants. After treatment for 14 days, we measured H_2_O_2_ content as well as the amount of cotyledon abscission. We observed a decrease in H_2_O_2_ content by 34% in the 5 mM AsA + 1 mM Zr treated seedlings and a decrease of 34% in the 5 mM DMTU + 1 mM Zr treated seedlings when compared to the Zr-only treated seedlings (Table 3). Again, the Zr treatment led to an increase in abscission of more than ~ 60%. But, when we applied the scavengers respectively, we observed no cotyledon abscission in the combination treatments (Table 3).

**Table 3:** H_2_O_2_ content and cotyledon abscission in *R. sativus* seedling shoots in response to Zr, AsA + Zr, DMTU + Zr treatments.

Trait	1 mM Zr	5 mM AsA + 1 mM Zr	5 mM DMTU + 1 mM Zr
H_2_O_2_ content [(nmol g^−1^ fresh weight (FW)]	760.15 ± 77.25^a^	502.10 ± 52.28^b^	505.30 ± 50.55^b^
Cotyledon abscision (%)	65.00 ± 6.00^a^	0.00 ± 0.00^a^	0.00 ± 0.00^a^

Different letters (a and b) per row indicate the means (± SE) that are significantly different at *p* < 0.05 (Tukey–Kramer test).

### MG reduces MDA and CD in seedlings under Zr stress

**Figure 3:**
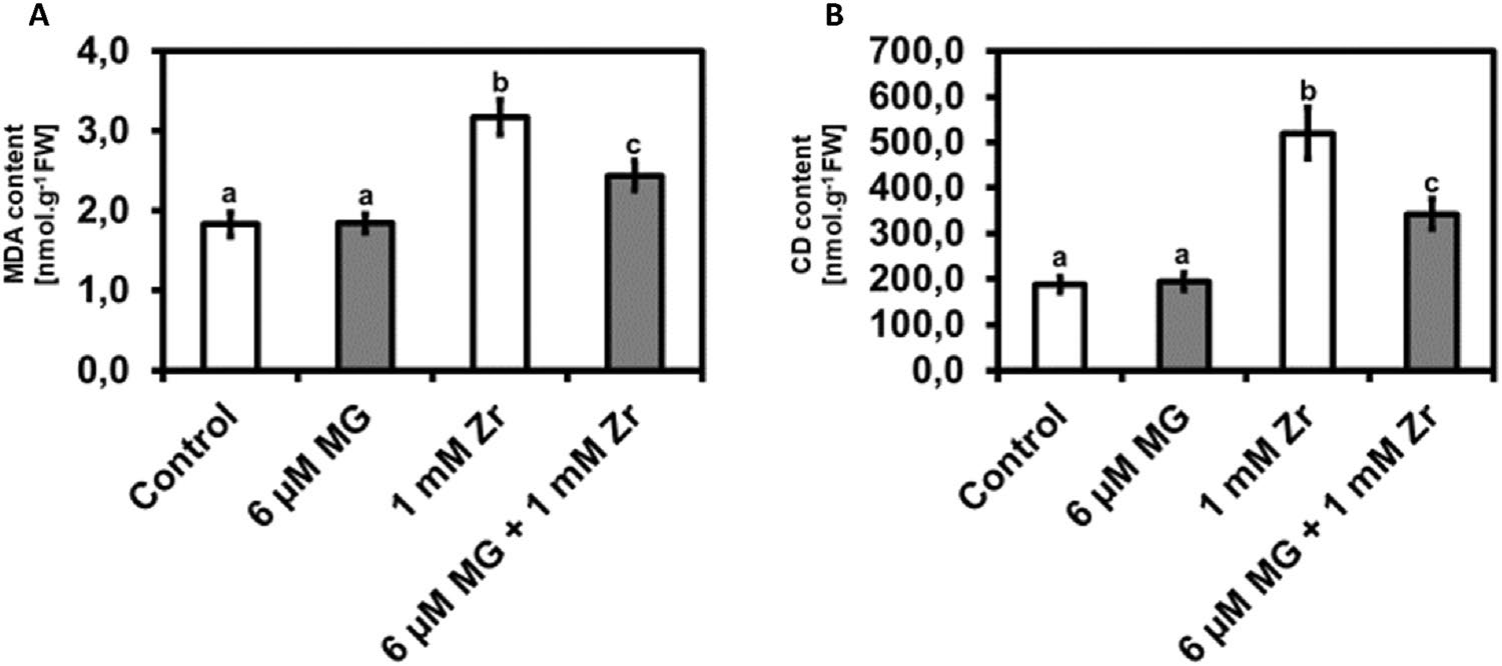
Malondialdehyde (MDA) content (**A**) and conjugated diene (CD) content (**B**) in *R. sativus* seedling shoots under control, MG, Zr and MG + Zr treatments. Different letters (a, b and c) represent the means (± SE) with statistical significance at *p* < 0.05 (Tukey–Kramer test).

After observing changes in the concentrations of reactive compounds (O_2_^−^, H_2_O_2_, **·**OH and MG) in response to the different treatments we then measured the concentrations of other lipid peroxidation-biomarkers namely MDA and CD. We observed no significant difference in MDA content in MG-treated seedlings when compared to the control. We observed increases in MDA content of 73% and 33% in the Zr treatment and the MG + Zr combination treated seedlings, respectively (Fig. 3A). We noticed a decrease in MDA content of 23% in the MG + Zr treatments when compared to the Zr-treated seedlings (Fig. 3A). We observed no significant difference in the CD content of the MG-treated seedlings (Fig. 3B) compared to the control. We noticed increases of 177% and 82% in CD content in the Zr treatment and in the MG + Zr combination-treated seedlings, respectively (Fig. 3B). The MG + Zr treatment led to a 34% decrease in CD content when compared to the Zr-treated seedlings (Fig. 3B).

### Exogenous MG increase SOD activity and increase APX activity in seedlings under Zr stress

**Figure 4:**
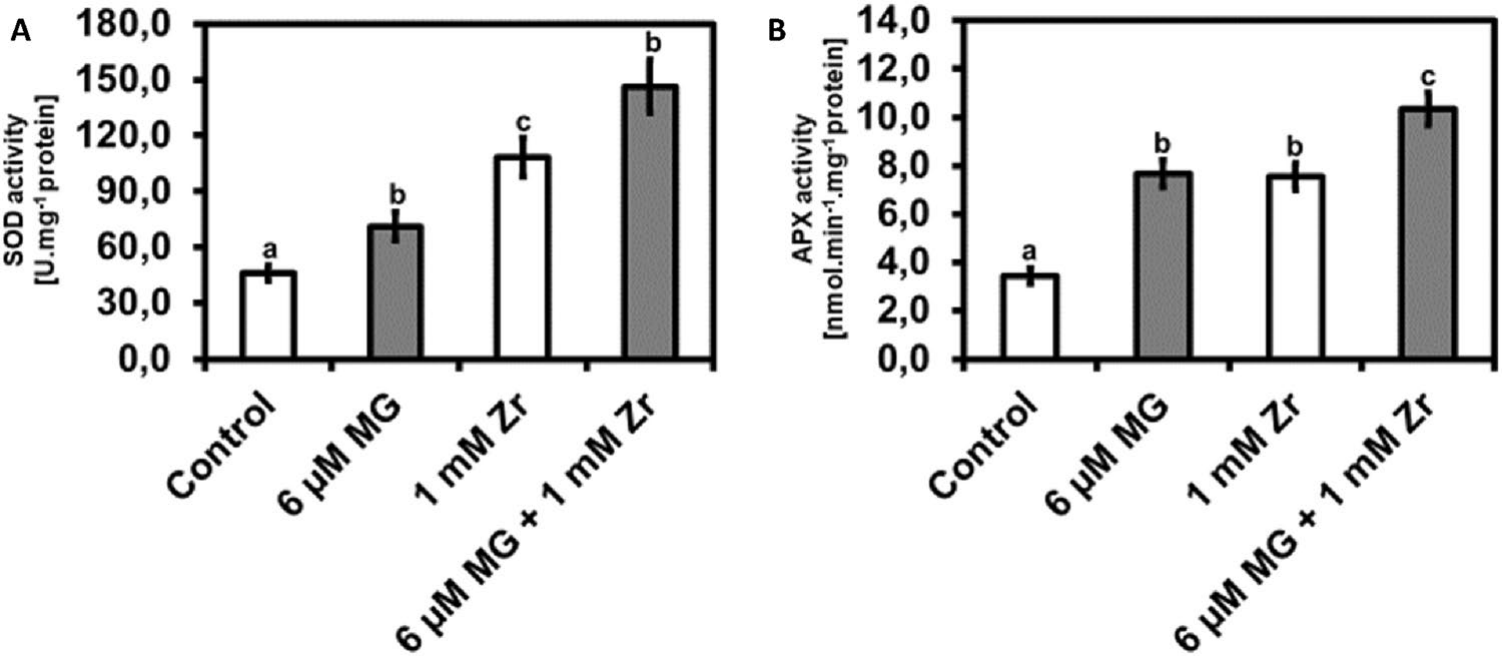
Superoxide dismutase (SOD) activity (**A**) and ascorbate peroxidase (APX) activity (**B**) in *R. sativus* seedling shoots under control, MG, Zr and MG + Zr treatments. Different letters (a, b, c and d) represent the means (± SE) with statistical significance at *p* < 0.05 (Tukey–Kramer test).

After observing changes in O_2_^•−^ and H_2_O_2_ content, we biochemically measured the SOD and APX activity in treated *R. sativus* seedlings. When compared to the control, we observed increases in SOD activities in MG-treated seedlings (53%), Zr-treated seedlings (135%), and MG + Zr combination-treated seedlings (218%), respectively (Fig. 4A). However, we observed an increase in SOD activity of 35% in the MG + Zr combination treatment when compared to the Zr-treated seedlings (Fig. 4A). Furthermore, compared to the control we observed increases in APX activities in MG-treated seedlings (123%), Zr-treated seedlings (120%), and MG + Zr combination-treated seedlings (201%), respectively (Fig. 4B). In addition, an increase in APX activity of 37% was observed in MG + Zr treatment when compared to the Zr-treated seedlings (Fig. 4B).

### Exogenous MG increases Gly I activity under Zr stress

**Figure 5:**
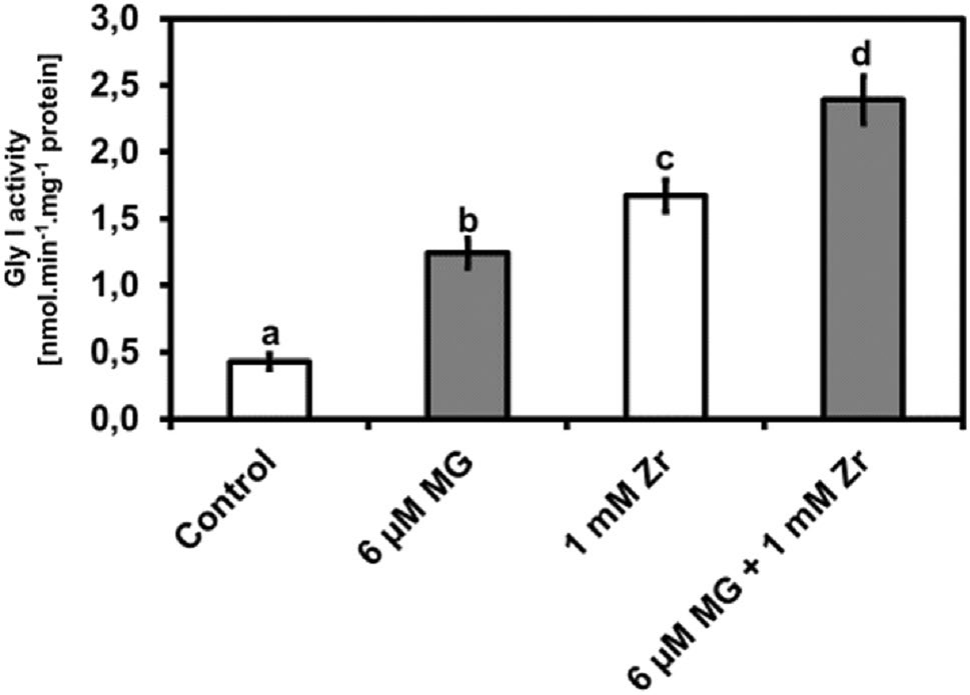
Gly I activity in *R. sativus* seedling shoots under control, MG, Zr and MG + Zr treatments. Different letters (a, b, c and d) represent the means (± SE) with statistical significance at *p* < 0.05 (Tukey–Kramer test).

After noticing changes in endogenous MG content in response to the treatments, we biochemically measured the activity of the MG scavenging enzyme Gly I in the *R. sativus* seedlings. Compared to the control, we observed increases in Gly activities in MG-treated seedlings (188%), Zr-treated seedlings (288%), and MG + Zr combination-treated seedlings (456%), respectively (Fig. 5). Furthermore, an increase in Gly I activity of 43% was observed in MG + Zr treatment when compared to the Zr-treated seedlings (Fig. 5). This finding shows that exogenous MG leads to increases in endogenous MG which induces Gly I activity in *R. sativus*. It also shows that Zr treatment leads to an increase in Gly I induction and that the combination triggers the highest Gly I induction in *R. sativus*.

### Exogenous MG reduces seedling shoot cell death under Zr stress

**Figure 6:**
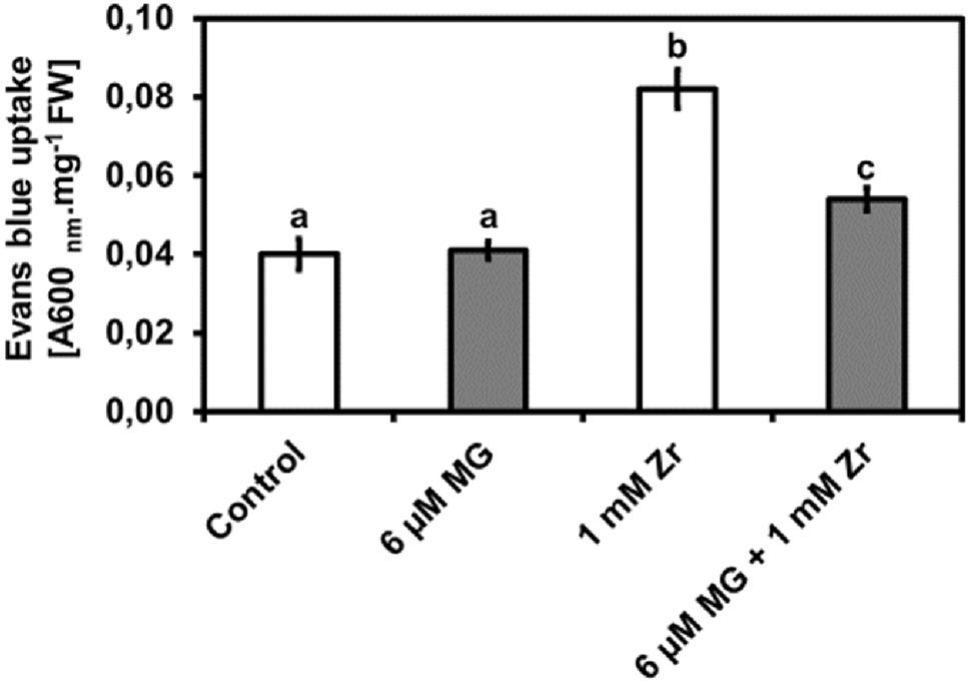
Seedling shoot Evans blue uptake (cell death) after control, MG, Zr and MG + Zr treatments. Different letters (a, b and c) represent the means (± SE) which differ significantly at *p* < 0.05.

Since we noticed changes in reactive compounds (O_2_^−^, H_2_O_2_, **·**OH and MG) as well as lipid peroxidation-biomarkers (MDA and CD), we measured cell membrane integrity and cell viability using Evans blue. Compared to the control, we observed no change in Evans blue uptake in MG-treated seedlings, an increase of 100% in Evans blue uptake in Zr-treated seedlings, and an increase of 25% in Evans blue uptake in the MG + Zr combination-treated seedlings, respectively (Fig. 6). Furthermore, we observed a decrease of 38% in Evans blue uptake in the MG + Zr treatment when compared to the Zr-treated seedlings (Fig. 6). This finding shows that exogenous MG does not lead to any cellular damage in *R. sativus*. It also shows that Zr treatment leads to an increase in cell damage and cell death. We speculate that the cellular damage is reversed when treating *R. sativus* seedlings with MG under Zr stress.

### MG reduce seedling root and shoot Zr content and the translocation factor under Zr stress

We measured the Zr content in the *R. sativus* seedling roots and shoots following treatments. No significant difference in Zr content was observed in MG-treated roots and shoots when compared to the control (Table 4). An increase in Zr root content of 152,421% and shoot content of 44,868% was observed in Zr-treated seedlings. An increase in Zr root content of 77,955% and shoot content of 8119% was observed in MG + Zr -treated seedlings when compared to the control. We also observed a decrease in Zr root content of 49% and shoot content of 82% in the MG + Zr combination treatment as compared to the Zr-only treatment (Table 4). Furthermore, TF analysis showed that exogenous application of MG did not change the TF, but Zr led to a 63% decrease in TF when compared to controls. We also observed a decrease in TF of 71% in the MG + Zr treatment when compared to the Zr-only treatment (Table 4).

**Table 4:** Total Zr content (µg g^−1^ FW) and Zr TF in *R. sativus* after control, MG, Zr, MG + Zr treatments.

Plant material/trait	Control	6 µM MG	1 mM Zr	6 µM MG + 1 mM Zr
Roots	2.83 ± 0.22^a^	2.82 ± 0.23^a^	4316.33 ± 404.45^b^	2208.96 ± 102.89^c^
Shoots	0.53 ± 0.04^a^	0.53 ± 0.04^a^	238.33 ± 24.54^b^	43.56 ± 4.56^c^
TF	0.19 ± 0.02^a^	0.19 ± 0.04^a^	0.07 ± 0.01^b^	0.02 ± 0.01^c^

Data represent the means (± SE) and different letters (a, b and c) per row are significantly different at *p* < 0.05 (Tukey–Kramer test).

## Discussion

MG is a signaling molecule which improve plant performance under abiotic stresses^17,24^ therefore to select the proper MG concentration to alleviate Zr stress in our study, we tested various MG concentrations (without Zr) in order to select the optimum concentrations for the Zr alleviation study. We observed improved seed germination at 2 µM, 6 µM and 10 µM but a decrease in seed germination at 100 µM and 200 µM (Supplementary material; Table S1). Furthermore, the 2 µM MG treatment did not improve the seedling dry weight but we observed an increase in dry weight at 6 µM and 10 µM MG. We noticed a decrease in seedling dry weight at 100 µM and 200 µM which might suggest that these concentrations lead to toxic effects in *R. sativus*. This suggestion is supported by the MG content result at 100 µM and 200 µM which shows excessive MG content in the seedlings (Supplementary material; Table S2). This finding is supported by Li et al.^25^ where MG improved seed germination of *T. aestivum* in a concentration dependent manner without stress treatment. For the alleviation study, we applied only the MG concentrations which improved seed germination and biomass (2 µM, 6 µM and 10 µM) under 1 mM Zr in a germination assay based on results from (Supplementary material; Figure S1). We observed the best germination performance at 6 µM MG and no effect at 10 µM MG which pointed to a MG concentration dependent seed germination improvement in *R. sativus* under Zr stress (Supplementary material; Figure S2). A similar result was also observed by Li et al.^25^ where MG improved seed germination of *T. aestivum* in a concentration dependent manner under salinity stress. We suspected that endogenous MG levels might play a role in our observations and conducted MG content assays on the seedlings. We did not observe any changes in MG content at 2 µM + 1 mM Zr treatment which explains why 2 µM could not improve seed germination. In contrast, the 10 µM MG treatment increased the MG levels in the MG + Zr treatment leading to no improvement of seed germination (Supplementary material; Table S3).

Visual inspection following heavy metal treatment is used to assess the impact of heavy metal stress on seedling growth^26,27^. In our study, we observed that MG increased *R. sativus* seedling shoot growth. Li et al.^28^ also observed an increase in *T. aestivum* growth in response to MG alone*.* These results show that MG improve seedling growth under normal conditions. This increase could be because of a direct effect of MG on cell size control or indirect effect through stimulating signaling molecules which control cell growth. Both processes have not been extensively investigated for plant MG and require further experimental investigation. We also observed restricted seedling shoot growth when the plants were treated with Zr which is in agreement with the results of Fodor et al.^16^. In that study seedling shoot growth was stunted in *T. aestivum*, following Zr treatment^16^. Furthermore, we observed improved plant growth in seedlings treated with MG + Zr and hypothesized that MG regulates signaling pathways by priming the adaptive response of the *R. sativus* seeds to the Zr stress which allowed the plants to cope under stress conditions. Li et al.^22^ also observed improvement of *T. aestivum* seedling growth under Cd toxicity and highlighted that MG alleviates Cd toxicity in the growing seedlings.

Plant biomass is reduced under heavy metal treatment^14^. In this study, Zr treatment led to a decrease in seedling shoot biomass which agreed with the observations of Simon et al.^29^ which showed that *Chlorella pyrenoidosa* biomass decreased in response to Zr treatment*.* Furthermore, MG only treatment led to an increase in shoot biomass and improved seedling shoot growth even in the presence of Zr which is supported by the study of Li et al.^28^ who also observed an increase in *T. aestivum* dry weights in response to MG + Cd stress*.* We suspected a direct link between biomass improvement and chlorophyll maintenance in response to MG and Zr. Wilkinson et al.^30^ have shown previously that a correlation exists between chlorophyll content and biomass in plants. In our study, we observed an increase in chlorophyll *a* and total chlorophylls in response to MG treatment. MG treatment did not change the levels of chlorophyll *b* in our study. Our result was supported by Majláth et al.^31^ who observed an increase in total chlorophylls in response to MG treatment in *T. aestivum*. We hypothesize that MG directly interacts with chlorophyll *a* and this could be concentration dependent however this notion requires further investigation. Saito et al.^32^ showed that MG directly interacts with chloroplasts but the effect of MG on individual chlorophyll species was not answered in that study. Zr treatment reduced the chlorophyll *a*, chlorophyll *b* and total chlorophyll levels in *R. sativus*. A reduction in chlorophyll *a* and chlorophyll *b* content was also observed by Simon et al.^29^ in *C. pyrenoidosa* in response to Zr stress. In addition, a reduction in total chlorophyll levels was also observed in *T. aestivum* by Fodor et al.^16^ under high Zr concentrations. When we treated seedlings with MG + Zr, we observed an increase in chlorophyll *a*, chlorophyll *b* and total chlorophyll levels in comparison to the Zr-only treatment. Our result is supported by the findings of Majláth et al.^31^ who showed that exogenous MG increased chlorophyll content to improve *T. aestivum* growth under cold stress. Therefore, we hypothesize that the chlorophyll *b* improvement by MG under the Zr stress could be as an indirect stress only regulation which is only induced under stress conditions. In a previous study, we also observed the same indirect regulation of chlorophyll *b* by signaling molecule 3,3′-diindolylmethane (DIM) under vanadium (V) stress in *B. napus*^18^ which supports our hypothesis. The increase in Zr content could have led to a reduction of magnesium (Mg) ions which could also explain the reduction observed. Then in this study, MG could improve the levels of Mg under Zr stress which leads to better production of chlorophylls since Mg is crucial for chlorophyll production. In addition, Zr could directly eliminate Mg from the chlorophyll structure and form a Zr-metaloporphirin however this needs further investigation. Zr could also lead to the inhibition of the chlorophyll synthesis enzymes: chlorophyll *a* oxidase, chlorophyll *b* reductase, 7-hydroxymethyl chlorophyll dehydrogenase, and magnesium-chelatase. This inhibition could be reversed by MG however this mechanism also needs further investigation.

Heavy metal toxicity lead to the generation of toxic reactive molecules in plants, which are not only harmful but they also have the ability to play signaling roles at low levels^28,33^. For example^34^, observed a negative growth response in exogenously H_2_O_2_ (5 mM) treated Arabidopsis where^35^ observed a positive growth response when treating Arabidopsis plants with 0.05 mM H_2_O_2_. In plants, ROS is produced from the reduction and oxidation of oxygen. More specifically, oxygen reduction leads to O_2_^•−^ production which is enzymatically converted to H_2_O_2_. Molecules of H_2_O_2_ is also formed by the two-electron oxidation of water and is subsequently oxidized to O_2_^•−^ and reduced to **·**OH^36^. In this study we measured the concentration of ROS (O_2_^•−^, H_2_O_2_ and **·**OH) as well as the reactive dicarbonyl compound MG in response to exogenous MG and Zr. MG treatment led to an increase in O_2_^•−^, H_2_O_2_ and MG content and no change in **·**OH content. It is known that MG treatment leads to a direct increase in O_2_^•−^ and H_2_O_2_ concentrations in animal systems^37^. In addition, MG binds to hemoglobin which releases iron that indirectly increases **·**OH concentration through the Fenton reaction in human cells^38^. However, the concentrations of O_2_^•−^, H_2_O_2_ and **·**OH have never been measured in MG-only treated plants. Nonetheless, we hypothesize that MG increases O_2_^•−^ and H_2_O_2_ for signaling purposes and our hypothesis is supported by Gokul et al.^18^ which observed increases in O_2_^•−^ and H_2_O_2_ but not **·**OH in *B. napus* in response to DIM. In our study, exogenous MG led to an endogenous increase in MG and this was also observed in *Zea mays* by Wang et al.^39^. Zr treatment led to an increase in the concentrations of O_2_^•−^, H_2_O_2_, **·**OH and MG in our study and this finding is supported by Fodor et al.^16^ in *T. aestivum* and Bless et al.^17^ in *Brassica rapa*. We also observed widespread cotyledon abscission in the Zr-only treated seedlings. Leaf abscission is an important stress response in plants because during stress periods plants can remove damage leaves in order to maintain proper energy balance^40^. We hypothesize that *R. sativus* remove damaged cotyledons under Zr stress in order to survive and this hypothesis is supported by Barceló and Poschenrieder^41^, which list leaf abscission as a response under heavy metal stress. Furthermore, Sakamoto et al.^42^ showed that H_2_O_2_ is involved in abscission signaling in *Capsicum baccatum* and *Capsicum chinense*. In that study, H_2_O_2_ promoted abscission in a dose-dependent manner and abscission was inhibited by the addition of a H_2_O_2_ scavenger, N-acetyl-L-cysteine (NAC)^42^. We used two H_2_O_2_ scavengers AsA and DMTU individually in combination with Zr and reduced the level of H_2_O_2_ in the *R. sativus* seedlings. Furthermore, similar to Sakamoto et al.^42^, we observed no abscission in the scavenger treated seedlings and this confirms our hypothesis that Zr induced abscission occurs via H_2_O_2_ signaling in *R. sativus*. Furthermore, the abscission observed in our study is also H_2_O_2_ concentration dependent since the scavengers decrease the H_2_O_2_ concentration to approximately the same level as the H_2_O_2_ concentration in the MG-only as well as the MG + Zr treatment. We postulate that MG treatment reduced the H_2_O_2_ content thus blocking abscission in the combination treatment. We also observed a decrease in the concentrations of O_2_^•−^, **·**OH and MG in the MG + Zr treatment when compared to the Zr-only treatments. This result proposes that MG decreased the content of reactive compounds which reduces toxicity and lowers direct interaction with the chlorophylls. This leads to improved photosynthetic metabolism and an improvement in biomass in *R. sativus* under Zr stress. Our hypothesis is supported by Mahmud et al.^43^, where the molecule GABA primed the scavenging enzymes which reduced all reactive compounds (O_2_^•−^, H_2_O_2_, **·**OH and MG) and improved the tolerance of *B. juncea* under Cr stress.

Lipid peroxidation is an unavoidable consequence of an increase in reactive compounds in plants. The levels of lipid peroxidation can be evaluated by determining the quantities of MDA and CD, respectively in response to heavy metal stress^44,45^. In our study, MG treatment did not change the levels of MDA and CD which shows that exogenous MG (at our study concentration) does not trigger direct changes in MDA and CD content, and this could be due to the unchanged **·**OH levels under MG treatment. Our postulate is supported by Gokul et al.^18^ which attributed the unchanged MDA and CD content under DIM treatment as a consequence of unaffected **·**OH levels in *B. napus*. Moreover, in our study, Zr treatment led to an increase in both MDA and CD content which agrees with the findings of Bless et al.^17^ in *B. rapa*. In addition, Zr treatment increased both MDA and CD levels and we attribute this to the increase in reactive molecules especially **·**OH. Our hypothesis is supported by Kaur et al.^46^ which showed that a direct link existed between **·**OH levels and MDA and CD content in Pb-treated *T. aestivum*. In our study, the MG + Zr treatment decreased the MDA and CD levels in comparison to the Zr-only treatments. We postulate that MG lowers the lipid peroxidation by reducing the reactive molecule content especially **·**OH content in *R. sativus* under Zr stress. A study by Ali et al.^47^ showed that lipid peroxidation decreases when the **·**OH content decreases. The authors highlighted that **·**OH initiates the lipid peroxidation reaction by removing hydrogen atoms from fatty acids in plants^47^. Kaur et al.^46^ supports our hypothesis by showing that sodium nitroprusside (nitric oxide donor) supplementation reduces MDA and CD contents in *T. aestivum* under Pb stress by decreasing the **·**OH content.

In plants, specialized enzymes scavenge reactive molecules to limit toxicity and reduce cell death under heavy metal stress^48^. Hence, we studied changes in total activities of SOD (O_2_^•−^ scavenger), APX (H_2_O_2_ scavenger), Gly I (MG scavenger) under MG and Zr treatments. MG treatment induced activities of SOD, APX and Gly I and this induction in enzymatic activity is due to the direct interplay between MG and scavenging enzymes^28^. In addition, SOD, APX and Gly I activities also increased in the Zr-treated seedlings which agreed with the findings of Bless et al.^17^ on *B. rapa*. The induction of scavenging enzymes under heavy metal stress is a well-studied defense mechanism in plants^14^. In our study, we detected increases in SOD, APX and Gly I activities in the MG + Zr treatment when compared to the Zr-only treatment. The ability to properly regulate antioxidant enzyme capacity under stress conditions improves plant growth and development^33^. Therefore, we hypothesize that the decreases in O_2_^•−^, H_2_O_2_, **·**OH and MG contents in response to the combination treatment is because of increases in the SOD, APX and Gly I activities in the MG + Zr treatment which is directly regulated by MG. Our postulate is supported by Wang et al.^39^ which observed that MG induced the activities of SOD, APX and Gly I which led to much better heat tolerance in *Z. mays*. We also hypothesize that MG could induce antioxidant enzymes through direct modification of transcription factors. Fu et al.^49^ observed direct modification of transcription factors in Arabidopsis under MG which supports our hypothesis however further investigation must be conducted in MG treated *R. sativus*.

Researchers use Evans blue dye to study changes in cell viability and to analyze membrane integrity in plants. Plant cells with damaged membranes will take up the dye and will stain blue^50^. Hence, here we measured Evans blue uptake and observed no change in dye uptake in the MG-only treatments which corresponds with the result observed in the study by Majláth et al.^31^. We also observed an increase in Evans blue dye uptake in the Zr-only treatments which agrees with the findings of Bless et al.^17^. Furthermore, we observed a reduction in dye uptake in the MG + Zr treatment when compared to the Zr-only treatment. Therefore, we hypothesize that MG treatment led to a reduction in toxic reactive molecules (O_2_^•−^, H_2_O_2_, **·**OH and MG) by directly inducing SOD, APX and Gly I activities under Zr stress. The resultant decrease in toxic molecules under MG treatment decreased the lipid peroxidation which in turn led to less membrane damage under Zr stress hence, we observed less dye uptake in the MG + Zr treatment than in the Zr-only-treated seedlings. Gokul et al.^18^ supports this hypothesis by observing an increase in SOD, APX, Gly I and GST under DIM and V combination stress and concluded that this resulted in less O_2_^•−^, H_2_O_2_, **·**OH and MG content as well as less lipid peroxidation which improved the cell viability of *B. napus* under vanadium stress.

Plants cope better under high heavy metal soil concentrations by regulating a plethora of different mechanisms. One mechanism is to circumvent metal absorption from the surroundings or to exclude the metals after the uptake process^51^. A second mechanism is to chelate or sequester metals which bypassed the initial exclusion step which prevents metal translocation from roots to shoots^52^. In our study, we measured the Zr content in *R. sativus* seedling roots and shoots following treatment with MG, Zr and MG + Zr treatments. The MG treatment led to no changes in Zr content in both roots and shoots compared to controls. We also observed no changes in the Zr translocation factor from roots to shoots following MG-only treatment. This result suggest that MG application does not change Zr uptake from control soil environments which agrees with observation of Gokul et al.^18^ where DIM treatment did not change the V uptake in *B. napus*. Furthermore, Zr treatment led to an increase in seedling root and shoot Zr content which corresponds to the observations made in a study by Fodor et al.^16^ where Zr treatment led to an increase in Zr content in *T. aestivum* seedling roots and shoots. We also observed a decrease in the Zr translocation factor from roots to shoots following the Zr treatment and which suggests that *R. sativus* actively blocks Zr translocation from roots to shoots as a survival mechanism and this observation is supported by Fodor et al.^16^. We also observed a reduction in Zr content in both plant tissues when we compared the MG + Zr treatment with the Zr-only treatment. Furthermore, the combination treatment led to a reduction in Zr translocation from roots to shoots when compared to Zr-only treated seedlings. Therefore, we hypothesize that MG suppress Zr transport only under Zr toxicity or stress conditions because MG does not act on Zr transport under normal conditions. The transporters for Zr in plants are not known^15^ but exogenous MG decreased the activity of the potassium transporter (KAT1) in *Arabidopsis thaliana*^53^, which proves that MG can directly regulate transporter activity in plants. Our hypothesis is also well supported because the signaling molecule hydrogen sulfide suppressed Cd uptake and translocation in *O. sativa*^54^ and aluminum uptake and translocation in *Hordeum vulgare*^55^ respectively, only under stress conditions.

## Conclusions

Our findings show that MG pretreatments can augment the Zr stress tolerance of *R. sativus* at the seed developmental stage. MG treatment reduced the movement of Zr from root to shoot in the combination treatment which suggests that MG could regulate Zr transporters in both roots and shoots. MG application increased enzymatic activities of SOD, APX, and Gly I under Zr stress which decreased the concentration of reactive compounds (H_2_O_2_, O_2_^*•−*^, MG and **·**OH). Lower levels of reactive compounds improved chlorophyll levels in the combination treatment in addition to a lowering the extent of lipid peroxidation (MDA and CD content). The observed lower lipid membrane damage led to a reduction in cell death which improved the seedling shoot growth and development under Zr stress. Zr stress led to an increase in cotyledon abscission, which was inhibited by MG through the regulation of H_2_O_2_ signaling via APX upregulation. This is the first study which connects MG to H_2_O_2_ signaling in plants under Zr stress. Our study highlights the potential signaling role of MG in the regulation of ROS under Zr stress at the early seedling development stage (summarized in Supplementary material; Figure S3). This study is only limited to the seedling shoot but future work could focus on non-soil experiments to analyze the root response by careful avoidance of root breakage. Further use of omics tools (transcriptomics, metabolomics, proteomics and ionomics) could identify crucial molecular, metabolite and elemental targets of MG in *R. sativus* plants and this could improve our understanding of MG signaling in the future.

## Materials and methods

### Plant growth experiments

The plants were propagated in a randomized design using a modified method of Gokul et al.^56^. The plants were grown in 15 cm diameter plastic pots which contained a nutrient rich potting mix (Stodels Nurseries, Cape Town, South Africa; 1-part Double Grow weed-free compost and 1 part Double Grow potting soil). The plants were grown under a day/night temperature cycle of 22 °C/16 °C with a light/dark cycle of 16/8 h and a day phase photosynthetic photon flux density of 300 μmol photons m^−2^ s^−1^. All experiments in this study comply with relevant institutional, national, and international guidelines and legislation. All methods that were not modified can be found in the Supplementary materials.

### Plant treatments

H_2_O for all experiments was purified by reverse osmosis (RO) followed by Milli-Q H_2_O purification [Millipore, Massachusetts, United States (USA)]. All control plants were treated with RO H_2_O only. For MG treatments, the RO H_2_O was supplemented with MG [MG solution; technical grade (~ 40% in H_2_O); Sigma, St. Louis, Missouri, USA]. For Zr treatments we supplemented the RO H_2_O with Zirconyl chloride octahydrate (ZrOCl_2_·8H_2_O; Sigma 98%). Three individual combination treatments were prepared as follows: (1) RO H_2_O was supplemented with MG and Zr simultaneously, (2) RO H_2_O was supplemented with the Hydrogen peroxide (H_2_O_2_) scavenger Ascorbic acid (AsA, Sigma) and Zr simultaneously, and (3) RO H_2_O was supplemented with the H_2_O_2_ scavenger Dimethylthiourea (DMTU, Sigma) and Zr simultaneously. Empty pots (no seeds) were pre-treated with the various treatments (100 mL) for 24 h. The next day 400 *R. sativus* seeds (Nooitgedacht cultivar was purchased from Agricol, Cape Town, South Africa;) were sowed per treatment. Germinated seeds and seedlings were treated twice a week for 14 days.

### Determination of Zr concentration for plant treatments

To determine the optimal Zr concentration to use in this study, we conducted a germination experiment over 14 days with 400 seeds per treatment using a modified method of Gokul et al.^18^. We determined the germination percentage of the *R. sativus* seeds by pre-treating empty pots (without seeds) with 100 mL of control (RO H_2_O), 500 µM Zr, 1 mM Zr and 2 mM Zr for 24 h. Then we planted all the seeds and observed the number of germinating seeds (seeds with radicals 3 mm or more in length), until no seeds germinated.

### Determination of MG concentration for plant treatments

To determine the optimal MG concentration to use in this study, we conducted a germination experiment and biomass assessment with 400 seeds per treatment using a modified method of Gokul et al.^18^. The seedless pots were first pre-treated with 100 mL of control (RO H_2_O), 2 µM MG, 6 µM MG, 10 µM MG, 100 µM MG and 200 µM MG for 24 h. The following day we planted all the seeds and observed the number of seeds that germinated up until no further seeds germinated. All treatments were applied for 14 days in twice per week intervals.

### Determination of MG concentration for Zr alleviation treatments

To determine the correct MG concentration for Zr alleviation in this study, we conducted a germination experiment with 400 seeds per treatment using a modified method of Gokul et al.^18^. Empty pots were pre-treated with 100 mL of control (RO H_2_O), 1 mM Zr, 2 µM MG + 1 mM Zr, 6 µM MG + 1 mM Zr and 10 µM MG + 1 mM Zr for 24 h. The following day we planted all the seeds and observed the number of seeds that germinated up until no further seeds germinated. All treatments were applied for 14 days in twice per week intervals.

### Preparation of plant material

After growing and treating the plants with control, 6 µM MG, 1 mM Zr, 6 µM MG + 1 mM Zr until the end of the seedling stage (14 days), we carefully separated shoots and roots. In order to prevent erroneous physiological and biochemical data interpretation as a result of root damage when the seedlings are removed from the soil, the roots were cleaned and only used in metal content analysis similar to the method by Gokul et al.^57^.

### Visual inspection and biomass (dry weight) assessment

The seedling shoots were visually inspected, and images were captured using a Canon 80D digital camera (lens; Canon EF-S 10–18 mm f/4.5–5.6 IS STM). Dry weight analysis was performed by drying individual intact seedling shoots for 48 h at 55 °C. Following the drying period, the weights were captured as described by Gokul et al.^56^.

### Seedling cotyledon abscission scoring

On day 14, we observed and scored the total percentage of cotyledon abscission using the scoring method of Suttle and Hultstrand^58^. Seedlings with 1 or 2 abscised cotyledons were scored as abscised cotyledons and seedlings with no abscission (2 cotyledons present) were labelled intact seedlings.

### Spectrophotometric assays

Absorbance measurements (end-point or kinetic) were recorded with a FLUOstar Omega UV–visible spectrophotometer (BMG LabTech GmbH, Ortenberg, Germany) according to the manufacturer’s instructions.

### Cell viability assay using Evans blue dye

The method of Gokul et al.^56^ was used to assess cell viability.

### Chlorophyll content estimation

The method of Nxele et al.^59^ was used to estimate total chlorophyll concentrations in the *R. sativus* shoots.

### Determination of MDA content

The method of Zhang et al.^60^ was used to assess lipid peroxidation.

### CD content assay

We determined the CD content with a modified method by Chérif et al.^61^ using the extinction coefficient of 26.5 mM cm^−1^. We ground the intact seedling shoots in liquid nitrogen to a fine powder. Ground material (100 mg) was mixed with reagent 1 [3 mL of 99.9% (*v*/*v*) methanol containing 100 mM ethylenediaminetetraacetic acid (EDTA), 3 mL of 99.9% (*v*/*v*) chloroform (containing amylenes as stabilizers)]. Reagent 2 [3 mL of a solution containing 5 mM EDTA and 1% (*w*/*v*) sodium chloride (NaCl)] was added to the mixture followed by centrifugation at 4000 × *g* for 10 min (4 °C). We removed the chloroformic phase with nitrogen gas and the leftover residue was dissolved in chloroform (500 µL). The mixture was subjected to nitrogen gas for further drying and the remaining sample (50 µL) was dissolved in absolute ethanol (800 µL). We collected the supernatant (200 µL) and measured the absorbance at 234 nm.

### Hydrogen peroxide content determination

The method of Velikova et al.^62^ was used to quantify the hydrogen peroxide content using a H_2_O_2_ standard curve from absorbance readings at 390 nm.

### Superoxide content determination

The method of Gokul et al.^56^ was used to quantify the superoxide content.

### MG content determination

The MG content was determined with the method of Gokul et al.^18^ using a MG standard curve.

### Hydroxyl radical concentration determination

We used a modified method of Halliwell et al.^63^ to estimate **·**OH concentrations in seedling shoots with the extinction coefficient of 155 mM cm^−1^. We recorded the weights of intact seedlings, and the shoots were placed in 50 mL tubes. The shoots were completely covered in a solution of 10 mM phosphate buffer (pH 7.4) and 15 mM 2-Deoxy-D-Ribose. Then, the samples were incubated for 4 h at 37 °C and completely homogenized. The homogenate (0.7 mL) was mixed with 3 mL of 0.5% (*w*/*v*) TBA [prepared in 5 mM sodium hydroxide (2 mL) and 1 mL of 100% (*v*/*v*) glacial acetic acid] by brief vortexing. The reaction mixture was heated for 30 min at 100 °C followed by immediate cooling on ice for 10 min followed by centrifugation at 10,000 × *g* for 5 min. We measured the absorbance of the collected supernatant at 532 as well as 600 nm and determined the ·OH concentration.

### Protein extraction for spectrophotometric assays

Intact seedlings were ground in liquid nitrogen. The resulting fine shoot powders (200 mg) were mixed with 1 mL of polyvinylpyrrolidone (PVP) buffer (40 mM phosphate buffer (pH 7.4), 1 mM EDTA, 5% (*w*/*v*) PVP (MW = 40,000), 5% (*v*/*v*) glycerol in RO H_2_O) and completely homogenized. We used the RC DC Protein Assay Kit 11 (Bio-Rad Laboratories, California, USA) to measure the protein concentrations according to the manufacturers’ instructions.

### APX activity assay

The method of Asada^64^, was used to measure seedling shoot APX activities with the extinction coefficient of 2.8 mM cm^−1^.

### Total SOD activity assay

The method of Stewart and Bewley^65^, was used to measure the seedling shoot SOD activities.

### Gly I activity assay

We used the method of Chakravarty and Sopory^66^, to measure the Gly I activity.

### Inductively coupled plasma optical emission spectroscopy (ICP-OES) analysis

We used the method of Vachirapatama and Jirakiattikul^67^, to acid digest frozen shoot and root material of treated *R. sativus* plants before determining the Zr concentration with The Varian Vista Pro CCD simultaneous ICP-OES (Varian, Australia) with certified standards (Sigma; TraceCERT).

### Statistical analysis

Twenty individual *R. sativus* seedlings per treatment were used for O_2_^•−^ content, cell death, **·**OH content and shoot dry weight measurements. Forty seedling shoots were grouped in pools of 10 seedlings per treatment for all other experiments. For the ICP-OES experiments, 40 seedling shoots and roots were digested individually in pools of 10 seedlings per treatment and the translocation factor (TF) was estimated as described by Gokul et al.^57^. Statistical analysis was performed on data obtained from six independent experiments using the one-way analysis of variance (ANOVA) test as well as the Tukey–Kramer test (set to *p* < 0.05) in the GraphPad Prism 8.0.1 software (www.graphpad.com).

### Supplementary Information

Supplementary Information.

## Data Availability

The data presented in this study are available in this final published manuscript.
