# Supplementary material for: Methylglyoxal improves zirconium stress tolerance in Raphanus sativus seedling shoots by restricting zirconium uptake, reducing oxidative damage, and upregulating glyoxalase I
Source: Sci Rep. 2023 Aug 21;13:13618. doi: 10.1038/s41598-023-40788-0 (PMC10442447; doi:10.1038/s41598-023-40788-0)
Supplement: Supplementary file 1 — Supplementary Information. [file 41598_2023_40788_MOESM1_ESM.docx]

**Supplementary Materials**

**Table S1.** *R. sativus* seed germination and seedling shoot dry weight in response to control and various MG concentrations (2 µM, 6 µM, 10 µM, 100 µM and 200 µM). Different letters (a, b, c and d) per row indicate the means (± SE) that are significantly different at *p* < 0.05 (Tukey–Kramer test).

| **Trait** | **Control** | **2 µM MG** | **6 µM MG** | **10 µM MG** | **100 µM MG** | **200 µM MG** |
| --- | --- | --- | --- | --- | --- | --- |
| Germination [%] | 78.51 ± 5.19^a^ | 92.66 ± 6.11^b^ | 95.00 ± 4.90^b^ | 95.00 ± 5.00^b^ | 40.23 ± 4.40^c^ | 15.87 ± 1.38^d^ |
| Dry weight [g] | 0.08 ± 0.01^a^ | 0.09 ± 0.01^a^ | 0.13 ± 0.01^b^ | 0.12 ± 0.01^b^ | 0.04 ± 0.01^c^ | 0.01 ± 0.01^d^ |

**Table S2.** *R. sativus* seedling shoot MG content (µM.g^-1^ FW) in response to control and various MG concentrations (2 µM, 6 µM, 10 µM, 100 µM and 200 µM). Data represent the means (± SE) of six independent experiments and different letters (a, b, c, d, e and f) per row indicate the mean values that are significantly different at *p* < 0.05 (Tukey–Kramer test).

| **Control** | **2 µM MG** | **6 µM MG** | **10 µM MG** | **100 µM MG** | **200 µM MG** |
| --- | --- | --- | --- | --- | --- |
| 71.51 ± 6.59^a^ | 88.34 ± 7.91^b^ | 112.55 ± 11.43^c^ | 157.23 ± 14.87^d^ | 1276.87 ± 110.56^e^ | 2345.54 ± 233.12^f^ |

**Table S3.** *R. sativus* seedling shoot MG content (µM.g^-1^ FW) in response to control, 1 mM Zr, 2 µM MG + 1 mM Zr, 6 µM MG + 1 mM Zr and 10 µM MG + 1 mM Zr treatments. Data represent the means (± SE) of six independent experiments and different letters (a, b, c and d) per row indicate the mean values that are significantly different at *p* < 0.05 (Tukey–Kramer test).

| **Control** | **1 mM Zr** | **2 µM MG + 1 mM Zr** | **6 µM MG + 1 mM Zr** | **10 µM MG + 1 mM Zr** |
| --- | --- | --- | --- | --- |
| 70.98 ± 7.10^a^ | 200.32 ± 20.56^b^ | 189.54 ± 18.55^b^ | 145.99 ± 14.23^c^ | 302.67 ± 28.88^d^ |


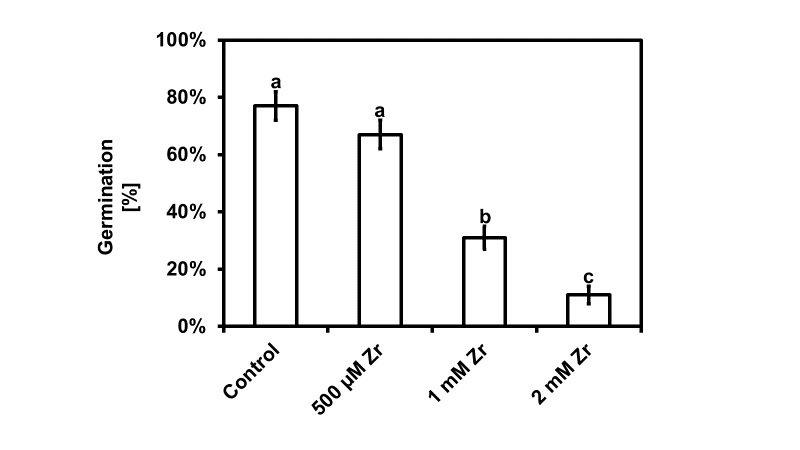


**Figure S1.** *R. sativus* seed germination in response to control, 500 µM Zr, 1 mM Zr and 2 mM Zr treatments. Different letters (a, b and c) indicate the means (± SE) that are significantly different at *p* < 0.05 (Tukey–Kramer test).


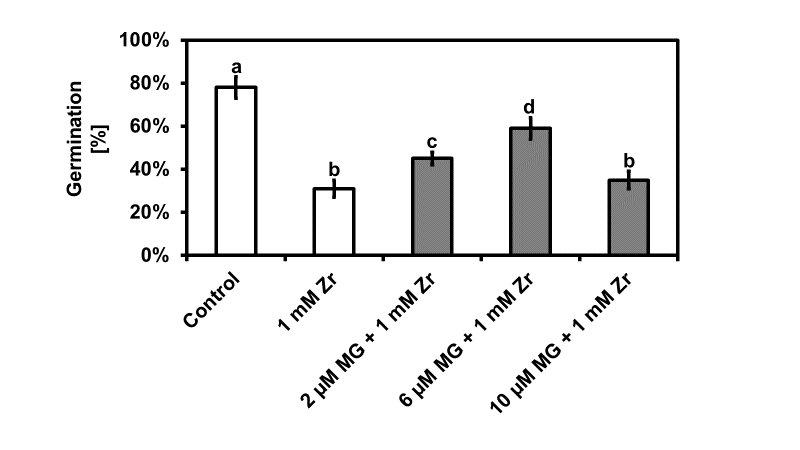


**Figure S2.** *R. sativus* seed germination in response to control, 1 mM Zr, 2 µM MG + 1 mM Zr, 6 µM MG + 1 mM Zr and 10 µM MG + 1 mM Zr treatments. Different letters (a, b, c and d) indicate the means (± SE) that are significantly different at *p* < 0.05 (Tukey–Kramer test).


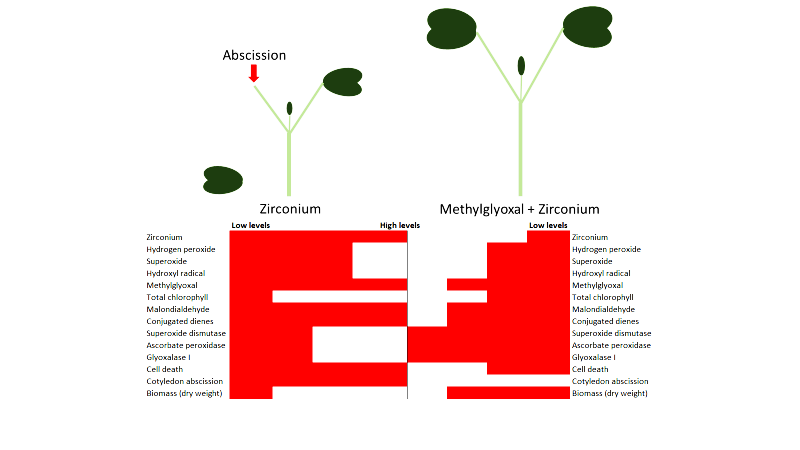


**Figure S3.** A cartoon figure showing arbitrary levels of measured parameters in response to zirconium and methylglyoxal + Zr combination treatments in *R. sativus* seedling shoots, respectively. Methylglyoxal application under zirconium treatment decreased most of the parameters measured accept for inducing an increase in total chlorophyll, superoxide dismutase activity, ascorbate peroxidase activity, glyoxalase I activity and shoot biomass.

Supplementary Materials and Methods

*Cell Viability Assay Using Evans Blue Dye*

The method of Gokul et al. ^57^ was used to assess cell viability. Briefly, the intact *R. sativus* seedling shoot was submerged in a 50 mL tube containing 0.25% (*w*/*v*) Evans blue (dye content ≥75%). The level of dye uptake was measured with a spectrophotometer at 600 nm.

*Chlorophyll Content Estimation*

The method of Nxele et al. ^60^ was used to estimate total chlorophyll concentrations in the *R. sativus* shoots. Briefly, we recorded the weight of the freshly harvested intact seedling shoots before homogenization in 5 mL of dimethylsulfoxide (DMSO) followed by incubation for 3 hours at 65 °C. The absorbance rates of the extract (200 μL) were measured at 645 nm and 663 nm, with DMSO as a blank.

*Determination of MDA content*

The method of Zhang et al. ^61^ was used to assess lipid peroxidation. Briefly, intact shoot material (100 mg) was ground into a fine powder using liquid nitrogen and homogenized in 1 mL of cold 6% (*w*/*v*) trichloroacetic acid (TCA). We mixed the supernatant (100 μL) with 400 μL of 0.5% (*w*/*v*) thiobarbituric acid [TBA; prepared in 20% (*w*/*v*) TCA] followed by incubation at 95 °C for 30 min. The reaction was placed on ice for 5 min (termination) and we centrifuged the mixture at 12,000× *g* for 5 min at 4 °C. The absorbance was recorded at 532 nm and 600 nm in order to subtract the non-specific absorbance. The MDA content was calculated from the absorbance readings with the extinction coefficient of 155 mM.cm^−1^.

*Hydrogen Peroxide Content Determination*

The method of Velikova et al. ^63^ was used to quantify the hydrogen peroxide content using a H_2_O_2_ standard curve from absorbance readings at 390 nm. Briefly, intact shoots were ground to fine powders in liquid nitrogen. The extracts (100 mg) was homogenized in 1 mL of cold 6% (*w*/*v*) TCA and centrifuged at 12,000× *g* for 30 min at 4 °C. Then, we mixed 50 μL of the supernatant with 5 mM dipotassium phosphate (K_2_HPO_4_, at pH 5.0) and 0.5 M potassium iodide (KI) in a total volume of 200 μL followed by incubation for 20 min at 25 °C.

*Superoxide Content Determination*

The method of Gokul et al. ^57^ was used to quantify the superoxide content. Briefly, intact seedling shoots were placed in a 50 mL tube, covered and incubated for 20 min in a mixture of 10 mM potassium cyanide (KCN), 10 mM H_2_O_2_, 2% (*w*/*v*) sodium dodecyl sulfate (SDS), 80 mM nitroblue tetrazolium (NBT) and 50 mM potassium phosphate buffer (pH 7.0). We homogenized the shoots and centrifuged the mixture at 10,000× *g* for 5 min. Then, we collected the supernatant and recorded the absorbance at 600 nm. We calculated the superoxide concentration with the NBT extinction coefficient of 12.8 mM.cm^−1^.

*MG Content Determination*

The MG content was determined with the method of Gokul et al. ^18^ using a MG standard curve. Briefly, intact seedling shoots (200 mg) were submerged in 2.5 mL 0.5 M Perchloric acid and homogenized. The resultant homogenate was incubated on ice for 15 min followed by centrifugation at 4 °C for 10 min at 11,000× *g*. We mixed the supernatant (1 mL) with activated charcoal (10 mg.mL^−1^) and incubated the mixture at room temperature for 15 min. We centrifuged the mixture at 11,000× *g* for 10 min and added saturated potassium hydroxide to the supernatant for neutralization at room temperature for 15 min. The mixture was centrifuged at 11,000× *g* for 10 min and we collected the neutralized supernatant. We mixed 650 μL of the supernatant with 330 μL of 100 mM Phosphate buffer (pH 7.0) and 20 μL freshly prepared 0.5 M N-acetyl-L-cysteine. The mixture was incubated for 15 min at room temperature and the absorbance was recorded at 288 nm.

*APX Activity Assay*

The method of Asada, ^65^ was used to measure seedling shoot APX activities with the extinction coefficient of 2.8 mM.cm^−1^. Briefly, we mixed the protein extracts (20 µL) with 2 mM L-AsA and incubated the mixture on ice for 5 min. The protein extract was mixed with the reaction mixture containing 50 mM phosphate buffer (pH 7.0), 0.1 mM EDTA and 50 mM L-AsA. We added 1.2 mM H_2_O_2_ to in a final volume of 200 μL to initiate the reaction and the APX activity was monitored kinetically at 290 nm.

*Total SOD Activity Assay*

The method of Stewart and Bewley, ^66^ was used to measure the seedling shoot SOD activities. The protein extract (10 μL) was briefly mixed by vortexing in 190 μL of the assay buffer [50 mM phosphate buffer (pH 7.8), 0.1 mM EDTA, 10 mM methionine, 5 μM riboflavin, 0.1 mM NBT]. We incubated the mixture under white fluorescent light at room temperature for 20 min. The absorbance was measured at 560 nm and calculated the SOD activity based on the enzymatic reduction of NBT (50% equals 1 Unit of SOD).

*Gly I Activity Assay*

We used the method of Chakravarty and Sopory, ^67^ to measure the Gly I activity. Briefly, we mixed 20 µL of the protein extracts with 180 µL of the reaction assay mixture [100 mM phosphate buffer (pH 7.5), 3.5 mM MG, 15 mM magnesium sulphate (MgSO_4_) and 1.7 mM glutathione (GSH)] followed by incubation at room temperature for 10 min. The Gly I activity was monitored spectrophotometrically (kinetic) at 240 nm.
